# Combining Pulsed Radiofrequency With Transcranial Magnetic Stimulation to Manage Hemiplegic Shoulder Pain and Somatosensory Nerve Transmissions

**DOI:** 10.1155/prm/9913659

**Published:** 2026-07-28

**Authors:** Ying Zhao, Xuehan Zang, Lingyan Wang, Zhengmei Yan, Jun Yang, Qingchuan Gu, Li Zhou, Xianwei Che, Aiqun Shi, Jiasheng Wang

**Affiliations:** ^1^ Department of Rehabilitation Medicine, Jinhua Hospital of Traditional Chinese Medicine Affiliated to Zhejiang Chinese Medical University, Jinhua, China; ^2^ Centre for Cognition and Brain Disorders, Affiliated Hospital of Hangzhou Normal University, Hangzhou, China; ^3^ Department of Pain Medicine, Ningbo No. 6 Hospital, Ningbo, China, nbdlyy.com

**Keywords:** hemiplegic shoulder pain, pain, pulsed radiofrequency, repetitive transcranial magnetic stimulation, stroke

## Abstract

**Background:**

Hemiplegic shoulder pain (HSP) is a major cause of disability that is still challenging to manage in clinical practices. Both pulsed radiofrequency (PRF) and repetitive transcranial magnetic stimulation (rTMS) could reduce poststroke HSP to certain extents. However, it remains unknown whether combined PRF and rTMS treatment could increase analgesic efficacy for poststroke HSP.

**Objectives:**

To evaluate the therapeutic efficacy of combined PRF and transcranial magnetic stimulation in treating HSP.

**Study Design:**

This was a randomized, assessor‐blinded pilot study.

**Setting:**

The rehabilitation department of a single hospital.

**Methods:**

In this pilot study, a 3‐arm study design was utilized to compare the treatment efficacy of combined PRF and rTMS treatment with a single PRF or rTMS strategy. Patients were randomly assigned to receive PRF, rTMS, or combined treatment. Outcomes assessments included pain intensity, motor activity, and neurophysiological activities.

**Results:**

Our data indicated that rTMS acted more efficiently to reduce pain than PRF treatment. Combined treatment had a larger long‐term efficacy than rTMS treatment alone. Both combined and single treatment improved motor recovery and neural transmissions, such as suprascapular nerve amplitude and somatosensory N20.

**Limitations:**

As we designed a pilot study, the sample size was small in each treatment arm. Our findings thus need to be validated in future large studies.

**Conclusion(s):**

These novel findings provide a safe and effective treatment strategy for poststroke HSP by combining PRF with rTMS treatment.

**Trial Registration:** Chinese Clinical Trial Registry: ChiCTR2500100181

## 1. Introduction

Hemiplegic shoulder pain (HSP) is the most common pain condition in stroke patients [1]. It is a major cause of disability after stroke, which could cause prolonged hospital stay, limited independence in activities, and decreased quality of life [2, 3]. A series of treatment strategies has been proposed to manage HSP, such as physiotherapy, functional electrical stimulation, and nerve blocks. However, it is still challenging to manage HSP in clinical practices [4].

Pulsed radiofrequency (PRF) over the suprascapular nerve is a novel treatment strategy for patients with HSP. Two studies have independently demonstrated the benefits of PRF for pain experiences and shoulder range of motion (ROM) [5, 6]. Another study further found a superior effect of PRF over nerve block with lidocaine in treating poststroke HSP [7]. However, one study indicated that PRF has limited effects for HSP, only demonstrating 20% response rate at 2‐month follow‐up [8]. These studies together highlight the potency of PRF in managing HSP as well as the necessity for further investigation.

In addition to PRF, repetitive transcranial magnetic stimulation (rTMS) is a noninvasive technology to induce neuroplastic changes [9–12]. In fact, rTMS has demonstrated consistent analgesic effects in neuropathic pain conditions [13–16]. In poststroke HSP, one study revealed a rapid decrease in pain experiences after rTMS compared to sham treatment [17]. However, another study did not find a superior effect of rTMS over sham treatment, but rTMS did have a time effect in pain interference and disabilities [18].

Given the potency of these two strategies, it is highly interesting to combine them to manage poststroke HSP. The current study was a pilot investigation of the analgesic efficacy of combined PRF and rTMS over each treatment strategy. It is hypothesized that each treatment would be able to decrease pain over time, but the combined PRF and rTMS strategy would improve efficacy compared to a single treatment. Findings from this investigation would open perspectives to optimize treatment efficacy in poststroke HSP.

## 2. Methods

### 2.1. Study Design

This was a randomized, assessor‐blinded pilot study. Eligible patients were randomly assigned to receive PRF, rTMS, or combined treatment. No sham or placebo control group was included because this was a feasibility pilot study. Pain assessments were conducted at baseline; posttreatment; and at 1, 3, and 6‐month follow‐ups, with participant recruitment occurring between November 1, 2023 and June 30, 2025. Other clinical and neurophysiological assessments were performed at baseline and posttreatment. An interim analysis was conducted after enrolling 6 participants per group (18 total), which represented a departure from the originally planned final sample size of 20 per group (60 total). With this sample size, the study was powered only to detect very large effects and was underpowered for small‐to‐moderate effects. Results should be interpreted as exploratory. Due to the invasive nature of PRF (needle insertion with local sensation) and the perceptible scalp tapping during active rTMS, it was not feasible to blind the treating physicians or the patients. However, outcome assessors were blinded to group allocation. All patients were informed about the study and provided written informed consent. This study was approved by the hospital’s ethics committee (2023031707).

### 2.2. Participants

**FIGURE 1:**
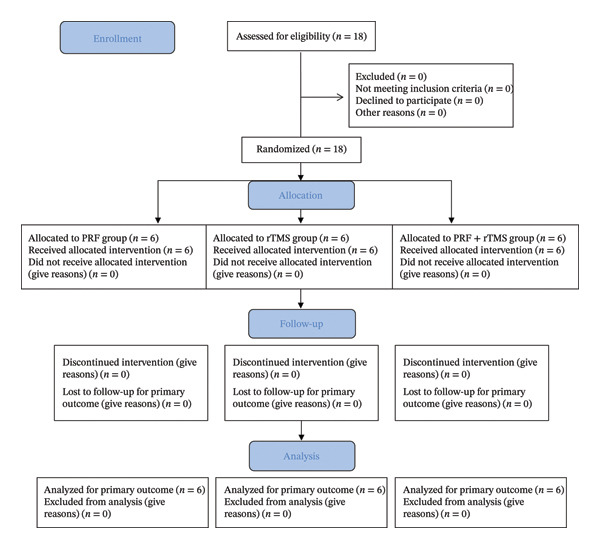
Participant recruitment flow diagram.

Potential participants were recruited from a certain hospital (Figure 1). The inclusion criteria were as follows: (1) unilateral cerebral insult due to stroke and hemorrhage; (2) at least 3 months after stroke; (3) absence of shoulder pain prior to the stroke; and (4) absence of peripheral neuropathies in the upper extremity prior to the stroke. The exclusion criteria were as follows: (1) contradictions to TMS treatment [19], such as metal implants or seizure; (2) aphasia or cognitive disorders (Mini‐Mental State Examination ≤ 24); (3) unwilling or unable to cooperate with treatment; (4) unstable medical condition or severe concurrent cardiovascular, pulmonary, hepatic, or renal diseases; (5) and history of substance abuse (alcohol and drugs). The withdrawal criteria included: (1) severe adverse reactions during the study; (2) development of severe complications or clinical deterioration during the study; (3) voluntary withdrawal by the participant.

### 2.3. PRF

All patients were treated by the same physician. Throughout the procedure, PRF was administered with the patient in a seated position. Prior to treatment, local anesthesia was achieved by injecting 2 mL of 2% lidocaine into the skin and subcutaneous tissues. Under ultrasound guidance (scanning frequency: 12 MHz), a 22‐gauge radiofrequency needle was advanced to the suprascapular notch. The suprascapular nerve was localized using anatomical landmarks and real‐time ultrasound visualization (trapezius, supraspinatus muscle, supraspinous fossa, and scapular spine), a well‐established approach for consistent nerve targeting in shoulder pain interventions [20]. After precise needle placement, sensory stimulation (50 Hz, 1 ms, 0.5 V) and motor stimulation (2 Hz, 1 ms, 0.4 V) were applied to elicit appropriate muscle responses to confirm accurate needle–nerve positioning [21]. PRF therapy was then delivered at 42 V, 20 ms, and 42°C for 300 s (https://www.bnsmedical.com/, Model R‐2000B M1). These parameters (42°C, 20 ms pulse width, 42 V, 5 min duration) were selected based on established clinical protocols for PRF of peripheral nerves, which prioritize neuromodulatory effects while minimizing thermal tissue damage [22].

### 2.4. rTMS

Resting motor threshold (RMT) was assessed in each session, which was the minimum intensity to induce motor evoked potentials (MEPs) larger than 0.05 mV in 5/10 trials. Surface electromyography was recorded from the first dorsal interosseous (FDI) muscle of the hand using 4 mm Ag/AgCl electrodes placed on the muscle in a belly–tendon arrangement. Single TMS pulses were delivered at a frequency of 0.2 Hz ± 10% over the primary motor cortex (M1) with a figure‐of‐eight coil (oriented at 45° away from the interhemispheric midline) connected to a Magstim Rapid^2^ system (Magstim Company Ltd, UK) [23]. This standardized procedure for RMT determination ensures consistent stimulation intensity across sessions and enhances the reproducibility of TMS interventions, consistent with established international guidelines for noninvasive brain stimulation in neurological populations.

All rTMS procedures were performed using the same figure‐of‐eight coil and magnetic stimulator as mentioned above. The 10‐Hz rTMS protocol consisted of 10 Hz trains for 5 s, with an intertrain interval of 55 s for a total of 1000 pulses [17]. The M1 corresponded to the motor hotspot of the affected hemisphere, which is the canonical target for neuromodulation in poststroke motor impairment and related pain conditions, as it directly modulates the corticospinal pathway and abnormal nociceptive processing [17]. The stimulation intensity was set to 90% RMT, an intensity commonly employed in poststroke populations to achieve neuromodulatory effects while minimizing adverse events and ensuring patient tolerability [17, 18]. Patients received 10 treatment sessions over 2 weeks [17, 18], a dosing schedule widely validated in studies of rTMS for poststroke motor and pain recovery.

### 2.5. Clinical Assessments

Outcome measures were reported according to the IMMPACT recommendations for chronic pain clinical trials [24]. The primary outcome was pain intensity measured on a 0–10 VAS (0 = no pain and 10 = maximal pain). A series of secondary clinical outcomes was also performed. The Fugl–Meyer Assessment for Upper Extremity (FMA‐UE) is a standardized, performance‐based scale designed to evaluate motor function, coordination, and reflexes in the upper limb after neurological injury [25]. We were interested in both the total score and the shoulder activity. Muscle strength refers to the maximal force‐generating capacity of a muscle or muscle group during voluntary contraction. It was quantified using the Medical Research Council ordinal scale (0–5). The Modified Ashworth Scale (MAS) was also used to assess muscle spasticity by grading resistance to passive movement on a 6‐point ordinal scale [26].

TMS safety assessments were performed at each treatment and follow‐up session. Headache and scalp discomfort were considered as mild side effects, which were most common in rTMS treatment [13]. Potential serious adverse effects were evaluated by monitoring patients’ vitality and physical and mental health.

### 2.6. Assessments of Neural Transmissions

Suprascapular nerve amplitude was assessed with the following parameters: bandpass filter = 20–3000 Hz, pulse width = 0.2 ms, stimulation frequency = 1 Hz, and sensitivity = 2000–5000 μV. The Erb point was stimulated with a supramaximal stimulation. The recording site was the supraspinatus muscle, where the compound muscle action potential (CMAP) amplitude was recorded using a concentric needle electrode. Both the ipsilateral and the contralateral sites were assessed.

For N20, the recording electrodes were positioned according to the international 10–20 EEG system, with C3 and C4 serving as the placement sites for contralateral median nerve stimulation. The latency of the N20 was recorded in both the ipsilateral and the contralateral sites.

### 2.7. Statistical Analyses

Demographic variables of patients were initially analyzed using one‐way ANOVA or *χ* ^2^ tests. Pain intensity was evaluated as the change in scores from baseline in the VAS. A mixed‐design ANOVA was then applied to VAS with group (3 levels) and time (5 levels). We then analyzed response rate, which was defined as a reduction in VAS score > 2 or > 30% [24–27]. We then compared the response rate with the *χ* ^2^ test. Suprascapular nerve amplitude, N20, and the Fugl–Meyer assessments were also expressed as the change scores from baseline. Mixed‐design ANOVAs were then applied to them with group (3 levels) and time (pre and post). As muscle strength and muscle spasticity were categorical variables, nonparametrical statistics were applied to these two assessments. Specifically, time effects and group effects were examined with the Wilcoxon and Kruskal–Wallis tests, respectively. In order to build up the relationship between outcome measures, correlations were performed with Pearson or Spearman tests where appropriate. All significant results were reported with *p* < 0.05. For pairwise comparisons, Bonferroni correction was applied within each outcome measure (3 comparisons per outcome), and the reported *p* values are the corrected ones as automatically output by SPSS (equivalent to raw *p* × 3).

## 3. Results

### 3.1. Demographic Information

Table 1 presents the demographic information of the patients. A total of 18 patients were enrolled and finished this study (6 in each arm). Among them, 10/18 were male patients, and the mean average was 62.11 years old. The average time since stroke was 4.83 months across groups, and most of the patients (12/18) had right‐hemisphere stroke. All these demographics were not different between the three groups.

**TABLE 1:** Demographic and general characteristics of participants.

	PRF group	rTMS group	PRF + rTMS group	*p* value
	*n* = 6	*n* = 6	*n* = 6	
*Demographics*				
Gender, *n* (%)				1.00
Male	3 (50%)	4 (66.7%)	3 (50%)	
Female	3 (50%)	2 (33.3%)	3 (50%)	
Age (years), mean ± SD	63.83 ± 13.42	59.50 ± 10.78	63.00 ± 11.70	0.81
Time since stroke onset (month)	5.5 ± 3.51	4.33 ± 1.51	4.67 ± 1.51	0.69
Affected hemisphere, *n* (%)				0.82
Left hemisphere	3 (50%)	2 (33.3%)	1 (16.7%)	
Right hemisphere	3 (50%)	4 (66.7%)	5 (83.3%)	
Comorbidities, *n* (%)				
Hypertension	6 (100%)	6 (100%)	6 (100%)	1.00
Diabetes mellitus	1 (16.7%)	1 (16.7%)	0 (0%)	1.00
Chronic kidney disease	0 (0%)	0 (0%)	0 (0%)	—
Depression	0 (0%)	0 (0%)	0 (0%)	—

*Note:* There were no significant differences in age, gender, disease duration, affected hemisphere, and comorbidities among the three groups.

### 3.2. Effects on Pain Intensity

**FIGURE 2:**
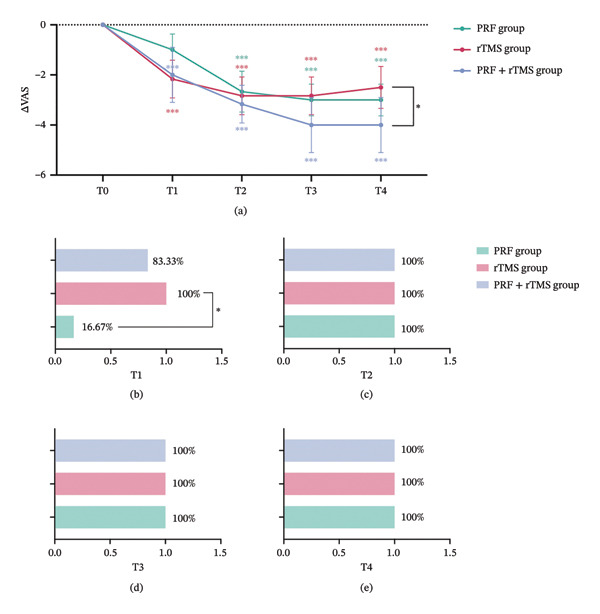
Treatment effects on pain scores and responder rates. (a) All groups demonstrated significant longitudinal reductions in VAS scores from baseline (PRF group‐T1: *p* = 0.11, T2–T4: *p* = 0.001; rTMS group‐T1–T4: *p* = 0.001; PRF + rTMS group‐T1–T4: *p* = 0.001). A significant between‐group difference was observed at T4 (rTMS vs. PRF + rTMS: corrected *p* = 0.029). (b) Responder analysis (≥ 30% VAS reduction or > 3‐point decrease) revealed significantly higher response rates in rTMS versus PRF group at T1 (immediately after treatment) (*p* = 0.015). (c) No significant intergroup differences in responder rates were observed among the three groups at T2 (1 month after treatment). (d) No significant intergroup differences in responder rates were observed among the three groups at T3 (3 months after treatment). (e) No significant intergroup differences in responder rates were observed among the three groups at T4 (6 months after treatment). ^∗^ *p* ≤ 0.05, ^∗∗^ *p* ≤ 0.01, and ^∗∗∗^ *p* ≤ 0.001.

Mixed‐designed ANOVA revealed an interaction in pain intensity (*F*_5.09,38.18_ = 4.89, *p* = 0.001, $\eta_{p}^{2}=0.40$). Post hoc testing revealed that the combined treatment induced a larger pain reduction than rTMS at 6‐month follow‐up (corrected *p* = 0.029). At posttreatment, only the PRF group did not show immediate analgesia (*p* > 0.05), whereas both the rTMS and combination groups did (*p*_*s*_ < 0.05). All three groups decreased pain from 1‐month to 6‐month follow‐up compared to baseline (*p*_*s*_ < 0.05) (Figure 2).

Response data indicated a significant difference in response rates among the three groups at posttreatment (Fisher–Freeman–Halton exact test, *p* = 0.012). Post hoc pairwise comparisons with Bonferroni correction showed that the PRF group had a significantly lower response rate than the rTMS group (corrected *p* = 0.045), while no other pairwise differences reached significance. In addition, all three treatments had a perfect response from 1‐month to 6‐month follow‐up (Figure 2).

### 3.3. Effects on Neural Transmissions

**FIGURE 3:**
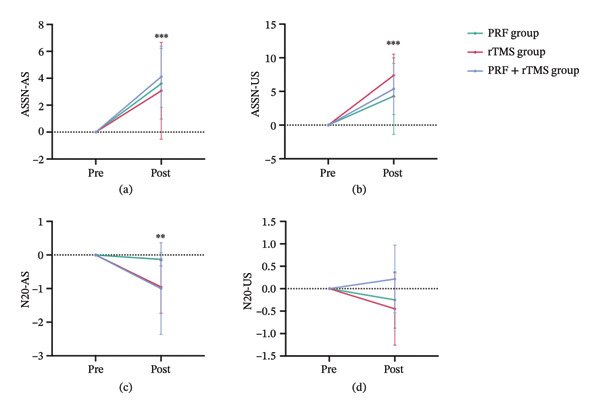
Treatment effects on suprascapular nerve amplitude and somatosensory N20 responses in affected versus unaffected sides. (a) In all three groups, the change in amplitude of the affected suprascapular nerve (ASSN‐AS) after treatment was significantly different from the baseline (*p* = 0.001). (b) The change in amplitude of the unaffected suprascapular nerve (ASSN‐US) after treatment was also significantly different from the baseline (*p* = 0.001). (c) The change in the N20 component of the somatosensory evoked potential on the affected side (N20‐AS) after treatment was significantly different from the baseline (*p* = 0.006). (d) The change in the N20 component on the unaffected side (N20‐US) after treatment was not significantly different from the baseline. ^∗^ *p* ≤ 0.05, ^∗∗^ *p* ≤ 0.01, and ^∗∗∗^ *p* ≤ 0.001. Abbreviations: ASSN: amplitude of the suprascapular nerve. N20: N20 component of somatosensory evoked potentials. AS: affected side. US: unaffected side.

For the ipsilateral suprascapular nerve, a mixed‐designed ANOVA revealed a time effect (*F*_1,15_ = 27.85, *p* = 0.001, $\eta_{p}^{2}=0.65$), suggesting that all three treatments increased suprascapular nerve amplitude after treatment. Similarly, all three treatments increased suprascapular nerve amplitude at the contralateral hemisphere (*F*_1,15_ = 30.70, *p* = 0.001, $\eta_{p}^{2}=0.67$) (Figure 3).

For somatosensory N20, a mixed‐designed ANOVA revealed a time effect on the ipsilateral hemisphere (*F*_1,15_ = 10.37, *p* = 0.006, $\eta_{p}^{2}=0.41$), suggesting that all three treatments decreased N20 latency after treatment. Meanwhile, no significant effect was found for the contralateral hemisphere (Figure 3).

### 3.4. Effects on Other Outcome Measures

**FIGURE 4:**
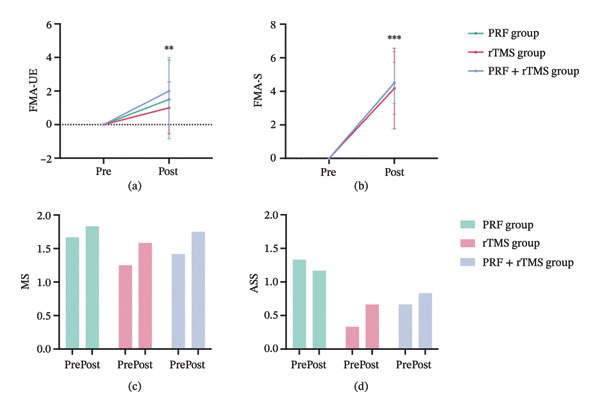
Treatment effects on motor function and spasticity. (a) After treatment, the changes in the Fugl–Meyer assessment of the upper extremity (FMA‐UE) were significantly different from baseline (*p* = 0.006). (b) Similarly, the changes in the Fugl–Meyer assessment of the shoulder (FMA‐S) after treatment were also significantly different from baseline (*p* = 0.001). However, no significant differences were observed in muscle strength (MS) (c) or the Ashworth Spasticity Scale (ASS) (d) before and after treatment. ^∗^ *p* ≤ 0.05, ^∗∗^ *p* ≤ 0.01, and ^∗∗∗^ *p* ≤ 0.001. FMA‐UE: Fugl–Meyer assessment of upper extremity. FMA‐S: Fugl–Meyer assessment of shoulder. MS: muscle strength. ASS: Ashworth spasticity score.

For upper extremity activity, a mixed‐designed ANOVA revealed a time effect (*F*_1,15_ = 10.21, *p* = 0.006, $\eta_{p}^{2}=0.41$), suggesting that all three treatments improved upper extremity recovery. The same time effect was found for shoulder motor recovery (*F*_1,15_ = 96.61, *p* = 0.001, $\eta_{p}^{2}=0.87$) (Figure 4).

For muscle strength, nonparametric statistics revealed that neither treatment was able to increase muscle strength (*p*_*s*_ > 0.05). There was also no effect for Ashworth’s cramp (*p*_*s*_ > 0.05) (Figure 4).

### 3.5. Correlational Results

**FIGURE 5:**
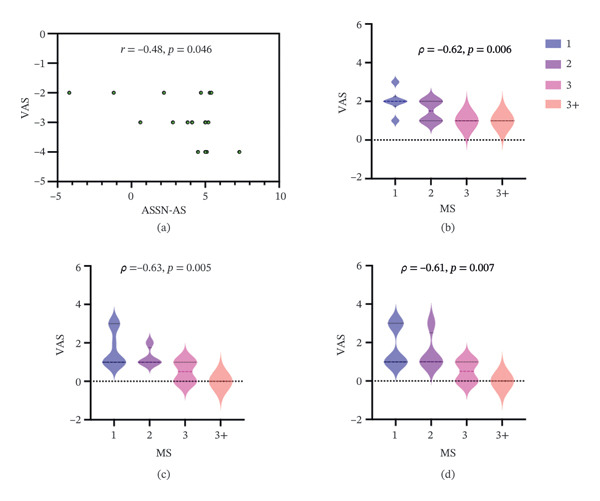
Correlation between VAS and motor or neurophysiological indicators. (a) The change in affected suprascapular nerve amplitude (ASSN‐AS) before and after treatment is significantly correlated with the change in VAS score at T2 (*p* = 0.046, *r* = −0.48). (b) Posttreatment muscle strength is significantly correlated with VAS score one month after treatment (*p* = 0.006, *p* = −0.62). (c) Posttreatment muscle strength is significantly correlated with VAS score three months after treatment (*p* = 0.005, *p* = −0.63). (d) Posttreatment muscle strength is significantly correlated with VAS score six months after treatment (*p* = 0.007, *r* = −0.61). ^∗^ *p* ≤ 0.05, ^∗∗^ *p* ≤ 0.01, and ^∗∗∗^ *p* ≤ 0.001.

Our data indicated that increased ipsilateral suprascapular nerve amplitude at posttreatment was associated with less pain at 1‐month follow‐up (*r* = −0.48, *p* = 0.046). In addition, posttreatment muscle strength was associated with less pain at 1‐month (*r* = −0.62, *p* = 0.006), 3‐month (*r* = −0.63, *p* = 0.005), and 6‐month (*r* = −0.61, *p* = 0.007) follow‐up (Figure 5).

### 3.6. Adverse Effects

No series side effects were reported in TMS treatment. Minor side effects were limited to mild headaches (0, 1, and 2 in the PRF, rTMS, and combined groups, respectively), resolving spontaneously within minutes or hours.

## 4. Discussion

This pilot study evaluated the efficacy of combining PRF with rTMS in managing poststroke HSP. Our data indicated a superior effect of combined treatment in managing poststroke HSP. Both combined and single treatments improved other symptoms, such as motor recovery and somatosensory neural transmissions. We also characterized the efficiency of different treatments where rTMS induced an immediate pain reduction.

Our data demonstrated that rTMS induced an immediate pain reduction after the completion of treatments as well as a long‐term effect lasting for 6 months. Meanwhile, either PRF or combined therapy was able to significantly decrease pain intensity at posttreatment. Previous consensus studies have identified Level A evidence of motor cortex rTMS in neuropathic pain but not in other pain conditions [15, 28]. Our findings indicated an immediate and long‐lasting effect on poststroke HSP, thus extending the clinical application of motor cortex rTMS for pain management. However, large, controlled trials are needed to further validate rTMS effects on poststroke HSP. It is worth noting that two previous studies provided inconsistent evidence of rTMS effects on HSP. One possibility is associated with rTMS frequency, in which 10 Hz trains induced significant analgesia, whereas 5 Hz did not [17, 18]. We used 10 Hz trains and generated consistent findings with the literature.

It is interesting to show that PRF over the suprascapular nerve acted slowly to reduce pain but was also able to generate long‐term analgesia. Specifically, PRF did not induce significant pain reduction 10 days after treatment and had a lower response rate than rTMS. One study indicated an immediate analgesic effect of PRF for HSP [5]. However, other studies reported clear analgesic effects starting at 1‐month follow‐up [6, 7]. Our data agree with the latter studies and demonstrate clear effects from 1 to 6 months posttreatment. Taken together with rTMS, these novel findings characterized the efficiency and efficacy of different treatment options for poststroke HSP.

More importantly, combining rTMS with PRF improved analgesic effects compared to single treatment. Specifically, combined therapy generated more pronounced pain reduction than rTMS at 6‐month follow‐up. Given the noninvasive nature of rTMS, this combined therapy provides a safe and effective treatment strategy for managing poststroke HSP. These interesting findings provide alternative options for managing poststroke HSP in contexts.

Mechanistic evidence may help understand the efficiency and efficacy of different treatment options for poststroke HSP. In rTMS‐induced analgesia, one prominent mechanism is the descending pain modulation [29]. For instance, the integrity of the thalamocortical tract was associated with the analgesic effects of M1‐rTMS [30, 31]. This was also supported by evidence that rTMS induced the release of endogenous opioids and decreased the pump attempts for anesthetics [32–35]. In addition, rTMS is suggested to modulate the affective structures of pain, such as the anterior cingulate cortex and insular cortex, to generate analgesia [36–38]. These multiple and potential parallel neural mechanisms may result in a quicker pain reduction in our data.

While our data revealed statistically comparable changes in ipsilateral somatosensory N20, PRF induced a less prominent reduction in N20 latency than the other two treatments. Although the underlying mechanisms remain unclear, PRF has been proposed to inhibit the transmission of nociceptive stimuli from the shoulder joint [39, 40]. Our findings may suggest that PRF is potentially less effective in improving somatosensory transmission from the median nerve. It is worth noting that all three treatments were equally effective in improving suprascapular nerve amplitude. Moreover, increased suprascapular nerve amplitude was associated with less pain experiences. These findings highlight a potential unique role of central or cortical transmission in generating a rapid analgesia in rTMS or combined therapy. However, given the small sample and multiple comparisons, we cannot draw mechanistic conclusions. Future studies with larger samples and dedicated mechanistic designs are warranted to test whether these changes mediate pain relief.

In addition, our data also revealed the effectiveness of all three treatments in motor recovery, including upper extremity and shoulder motor recovery. Although a time effect on muscle strength was not observed in individual group analysis, a significant improvement in muscle strength was identified when data were pooled across groups (*p* = 0.038). Moreover, our correlational data revealed that posttreatment muscle strength is consistently correlated with pain reduction at 1, 3, and 6 months following treatment. These positive results further corroborate our findings in pain perception and somatosensory transmission. Moreover, they are consistent with previous studies that indicated the benefits of rTMS or PRF treatment in motor recovery [8, 15, 41, 42]. It is worth noting that there were no benefits for Ashworth′s cramp in either treatment.

There were some limitations in this study. The primary limitation is the small sample size (*n* = 18). As a pilot study, it was not designed to provide definitive evidence of efficacy; accordingly, the observed statistically significant differences should be interpreted with caution, given the elevated risk of Type II and Type I errors. Our findings thus need to be validated in future large studies. Due to ethical considerations, this study was not able to design a control group that would ideally limit all the three active treatments. This limits our ability to differentiate treatment‐specific effects from placebo responses or spontaneous recovery, which is particularly relevant in poststroke populations where natural improvement can occur. Future large‐scale trials should incorporate a sham control group for both PRF and rTMS. Nonetheless, we designed two single treatments as active control and demonstrated a superior effect of our combined treatment. Another limitation is that the study was not truly double‐blind, which may introduce performance bias; future trials should consider more rigorous blinding strategies where possible.

In conclusion, our pilot study demonstrates that combining PRF with rTMS has potential benefits over single treatment in managing poststroke HSP. This effect also comes along with improvement of other symptoms, such as motor recovery and somatosensory neural transmissions. However, adequately powered, sham‐controlled trials are needed to confirm efficacy.

## Funding

This research was funded by the Key Projects in the Social Development Category of the Jinhua Science and Technology Research Plan (Grant no. 2023‐3‐142).

## Conflicts of Interest

The authors declare no conflicts of interest.

## Data Availability

The data that support the findings of this study are available from the corresponding authors upon reasonable request.
